# Les maladies rares et leurs manifestations cliniques orales dans deux formations hospitalières de Yaoundé

**DOI:** 10.11604/pamj.2019.32.195.14684

**Published:** 2019-04-22

**Authors:** Hubert Désiré Mbassi Awa, Rose Mbédé Nga Mvondo, Séraphin Nguefack, Charles Bengondo Messanga, Paul Olivier Koki Ndombo

**Affiliations:** 1 Centre Mère et Enfant de la Fondation Chantal Biya, Yaoundé, Cameroun; 2 Faculté de Médecine et des Sciences Biomédicales de l'Université de Yaoundé I, Cameroun; 3 Hôpital Gynéco-Obstétrique et Pédiatrique de Yaoundé, Yaoundé, Cameroun; 4 Centre Hospitalier et Universitaire de Yaoundé, Yaoundé, Cameroun

**Keywords:** Maladies rares, manifestations orales, soins bucco-dentaires, besoins spécifiques, Rare diseases, oral manifestations, oral care, specific needs

## Abstract

**Introduction:**

Les maladies rares ont pour certaines des manifestations orales. Celles-ci sont souvent sous-étudiées; ce qui contribue à limiter l'offre de soins bucco-dentaires pour cette catégorie de patients. Le but est de déterminer les aspects épidémiologiques et cliniques des manifestations bucco-dentaires dans les maladies rares dans notre milieu.

**Méthodes:**

Nous avons effectué une étude transversale et descriptive sur une durée de 7 mois, dans deux hôpitaux de référence de Yaoundé: les informations ont été recueillies des dossiers médicaux, de l'interrogatoire des parents ou tuteurs et de l'examen bucco-dentaire des patients. Le seuil de significativité considéré pour p < 0,05.

**Résultats:**

Les manifestations orales sur maladies rares sont variables et fonction du groupe d'affections, de la denture, et ont souvent un retentissement fonctionnel. Elles étaient présentes dans 97,2% de nos patients. En denture temporaire (59,4%), c'était plus des anomalies de forme et de position dentaire (conicité dentaire 7 cas/22, soit 32%), et en denture permanente des lésions carieuses (7 cas/10 soit 70%) et des anomalies de structure dentaire (4 cas/10 d'usure dentaire). Un lien significatif existait entre le type de denture, l'anomalie de structure (p=0,001) et de nombre (p=0,018). Les difficultés à la mastication (p=0,023) et à la succion (p=0,033) étaient liées aux groupes de maladies rares.

**Conclusion:**

Les lésions orales dans les maladies rares bien que présentes dans notre milieu, sont souvent négligées. Les soins bucco-dentaires devraient être intégrés dans le paquet minimum des activités des formations sanitaires et rendus gratuits ou financièrement accessibles à ces patients handicapés ou présentant des besoins spécifiques.

## Abstract (en)

**Introduction:**

Rare diseases have, in certain cases, oral manifestations. These are often understudied; this helps to limit oral care services to this category of patients. This study aims to determine the epidemiology and clinical features of oral manifestations in patients with rare diseases in our environment.

**Methods:**

We conducted a cross-sectional descriptive study in two referral hospitals in Yaoundé over a period of 7 months. Data were collected from patients' medical records, parents' or guardians' interview and oral examination. The threshold significance level was p< 0.05.

**Results:**

Oral manifestations associated with rare diseases vary based on the group of conditions, teeth and often have functional consequences. Functional impairment was diagnosed in 97.2% of cases. Patients with deciduous dentition (59.4%) most commonly developed anomalies of tooth shape and position (conicity 7/22 cases, 32%) while patients with permanent teeth most commonly developed carious lesions (7/10 cases, 70%) and anomalies in tooth structure (4/10 cases with dental wear). A significant link between type of tooth, anomalies in tooth structure (p=0.001) and tooth numbers was found (p=0.018). Mastication problems (p=0.023) as well as suction problems (p=0.033) were linked to rare disease patient groups.

**Conclusion:**

Oral lesions in patients with rare diseases, although present in our environment, are often neglected. Oral care should be integrated within the basic package of oral health care trainings after the establishment of free or affordable health care for patients with disabilities or with specific needs.

## Introduction

L'Organisation Européenne pour les Maladies Rares (EURORDIS) définit les maladies rares comme étant des affections invalidantes, touchant 1/2 000 personnes [1]. Ces affections sont d'hétérogénéité et de sévérité variables. Des éléments dysmorphiques sont parfois associés au tableau clinique et peuvent concerner le squelette facial ou mandibulaire, l'embryogénèse dentaire, ou l'ostéogenèse… Par ailleurs, des facteurs environnementaux ou épigénétiques, peuvent être déterminants à l'apparition de manifestations buccodentaires chez les patients atteints de maladies rares. Toutefois, en Afrique en général et au Cameroun en particulier, peu d'études font état de la fréquence d'observation de ces maladies et de leurs manifestations orales [2,3]. Pourtant, selon la base de données des dysmorphies de Londres portant sur 7000 maladies rares répertoriées, plus de 900 ont une composante dento-oro-faciale et 750 sont associées à des fentes labio-palatines [4]. Au Cameroun, dans une étude récente menée par Wonkam *et al.* en 2015 [5] et portant sur une série de cas de dystrophie musculaire de Duchenne, les auteurs avaient observé une dysmorphie linguale chez 2 sujets de leur série. Par ailleurs, l'errance diagnostique habituelle dans un contexte d'insuffisance de plateau technique, de stigmatisation des patients, de barrières aux soins, ou de l'inexistence d'un traitement spécifique, la prise en charge des cas est souvent retardée ou compromise. Ceci a justifié la dénomination de maladies orphelines pour la grande majorité de ces maladies [6]. Parmi les carences en soins à cette catégorie de patients, figurent en bonne place les soins bucco dentaires. C'est ce qui a inspiré la réalisation de ce travail, dont le but était d'étudier les aspects épidémiologiques et cliniques des manifestations bucco-dentaires chez des personnes vivant avec une maladie rare; afin d'évaluer le besoin et les types de soins à prévoir pour cette catégorie de patients souvent négligés.

## Méthodes

Nous avons mené une étude transversale et descriptive au sein des services de pédiatrie de deux formations hospitalières de référence à Yaoundé: l'Hôpital Gynéco-Obstétrique et Pédiatrique de Yaoundé (HGOPY) et le Centre Mère et Enfant de la Fondation Chantal Biya (CME-FCB). L'étude a été réalisée sur une période de sept mois, de novembre 2015 à mai 2016. Le choix de ces deux sites se justifiait par l'existence de consultations de neurologie pédiatrique (HGOPY et CME-FCB), et de Génétique (HGOPY). Par ailleurs, dans le cadre de la coopération entre le CME-FCB et l'Association ALMOHA (Association pour la lutte contre les maladies rares, les maladies orphelines et le handicap en Afrique), des semaines d'activités humanitaires sont souvent organisées conjointement, pour commémorer la journée mondiale contre les maladies rares. A ces occasions, l'expertise d'une équipe de généticiens français est souvent mise à contribution pour étayer certains diagnostics par des consultations sur sites et des tests génétiques à l'étranger, après consentement éclairé écrit des patients ou familles. L'échantillonnage était non probabiliste et consécutif. Était inclus dans notre étude, tout sujet atteint de maladie rare, inscrit dans les registres des formations hospitalières sus citées, se plaignant de symptômes buccaux ou non, et dont le parent ou tuteur avait donné son consentement éclairé pour la participation du patient à l'étude. Les patients décédés ou perdus de vue avaient été exclus de notre étude. Les registres de consultation avaient été exploités aux fins d'identification des patients porteurs de maladies rares. Nous avions ensuite contacté les parents ou tuteurs des patients par téléphone pour un entretien en tête à tête, au cours duquel des informations sur tous les aspects de l'étude leur étaient données. Leurs consentements éclairés verbal et écrit étaient recueillis et c'est alors que nous avions enfin procédé au recrutement des patients. L'assentiment du patient était également obtenu quand l'âge du patient et son état mental le permettaient. La suite de la procédure consistait en pratique en un interrogatoire et un examen physique détaillé ciblés. Les données collectées comportaient: les informations socio-démographiques notamment: l’âge, le sexe, le niveau d´instruction, le lieu de résidence et la région d´origine. Les antécédents familiaux de maladies similaires, les antécédents personnels d´affections bucco dentaires, de consultation de génétique ou de stomatologie. Puis un examen physique à la recherche de signes exo-buccaux et endo-buccaux était conduit. Les données recueillies pour chaque participant étaient consignées sur une fiche technique établie et pré testée. Les patients présentant des anomalies bucco-dentaires étaient orientés vers une consultation spécialisée. Les frais de déplacement des patients et de leurs parents ou tuteurs occasionnés par l'étude leur étaient restitués. Cette étude avait obtenu la clairance éthique du Comité Institutionnel d'Ethique et de Recherche de la Faculté de Médecine et des Sciences Biomédicales de l'Université de Yaoundé-I et celle du Comité Institutionnel d'Ethique de la Recherche pour la Santé Humaine de l'Hôpital Gynéco-Obstétrique et Pédiatrique de Yaoundé. De même, toutes les clairances administratives nécessaires avaient été obtenues. Les différents principes de la déclaration d'Helsinki avaient guidé toutes les étapes de cette étude, notamment la confidentialité, l'innocuité, le respect de la dignité et de l'intégrité des patients. Les informations collectées ont été saisies à l´aide d´un masque de saisie conçu à l´aide du logiciel CSPro 6.1. Elles ont été analysées grâce au logiciel SPSS version 18.0, les diagrammes, figures et tableaux ont été réalisés avec Microsoft Excel 2007. Le test de Chi carré nous a permis la comparaison de proportions, et de faire des corrélations. Une valeur p < 0,05 était considérée comme significative.

## Résultats

Parmi, les 40 patients que nous avions présélectionnés, trois avaient été exclus de notre étude et les 37 retenus avaient été examinés après obtention du consentement éclairé de leur parent ou tuteur.

Distribution de l'échantillon en fonction de l'âge, du sexe et du niveau d'instruction: notre échantillon comportait 19 filles (51,4%) et 18 garçons (48,6%). Soit un sexe-ratio garçons sur filles de 1:1,05. Les deux sexes sont donc pareillement concernés. L'âge des patients variait de 17 mois à 21 ans. La tranche d'âge la plus représentée était celle comprise entre un et quatre ans, avec un effectif de 20, soit 54,1%. Concernant le niveau d'instruction, 23 participants n'étaient pas scolarisés (62,2%), sept avaient un niveau d'instruction primaire (18,9%), six étaient au secondaire (16,2%) et un avait un niveau d'instruction universitaire (2,7%). Aucun patient ne suivait une éducation spécialisée.

Répartition de l'échantillon en fonction de la région d'origine: parmi les 37 participants, 19 étaient originaires de la région de l'Ouest (51,4%), sept de la région du Centre (18,9%) (Tableau 1). Aucun cas de maladie rare originaire des régions de l'Adamaoua, du Sud-Ouest ou encore d'un pays étranger n'a été observé.

**Tableau 1:** Répartition de la population en fonction de la région d’origine

Région d’origine	Nombre (N)	Pourcentage (%)
Centre	7	18,9
Littoral	1	2,7
Est	2	5,4
Ouest	19	51,4
Extrême Nord	2	5,4
Nord	1	2,7
Sud	3	8,1
Nord-Ouest	2	5,4
Sud-Ouest	-	-
Adamaoua	-	-
**Total**	**37**	**100,0**

**Figure 1:**
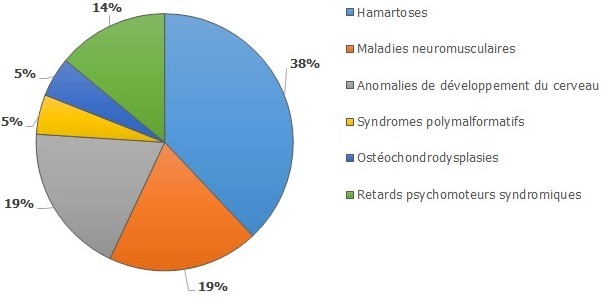
Grands groupes de maladies rares

Antécédents personnels et familiaux pertinents: seuls 3% de la parenté des patients étaient aussi atteints de la même maladie rare. Il s'agissait des cas d'ostéochondrodysplasie. Des consultations de génétique avaient été faites chez 22% des patients. Alors que 14% seulement des sujets avaient déjà bénéficié d'un examen bucco-dentaire par un spécialiste de la sphère bucco-dentaire. Nous avons recensé 19 maladies rares au cours de notre étude. Celles-ci étaient réparties en six grands groupes notamment: les hamartoses, les maladies neuromusculaires, les anomalies de développement du cerveau, les retards psychomoteurs syndromiques, les syndromes polymalformatifs et les ostéochondrodysplasies (Figure 1). Le groupe des hamartoses étaient le plus représenté avec 14 cas sur 37 (38%). La distribution spécifique des maladies rares de notre échantillon était comme suit: (Tableau 2).

**Tableau 2:** Distribution spécifique des maladies rares de notre échantillon

Groupe de maladies	Spécification des maladies	Effectif
**Hamartoses**	Sclérose tubéreuse de Bourneville	9
	Syndrome de Protée	3
	Syndrome de Sturge Weber	1
	Neurofibromatose de type I	1
**Maladies neuromusculaires**	Dystrophie musculaire de Duchenne	3
	Amyotrophie spinale infantile	2
	Dystonie déformans	2
**Anomalies de développement du cerveau**	Holoprosencéphalie	2
	Agénésie du corps calleux	1
	Lissencéphalie	1
	Encéphalocèle	1
	Kyste prosencéphalique	1
	Microcéphalie vera	1
**Retards psychomoteurs Syndromiques**	Syndrome de Rett	2
	Syndrome de Wolf Hirchhorn	1
	Syndrome de X-fragile	1
	Syndrome génétique et cardiaque	1
**Syndromes polymalformatifs**	Jumeaux conjoints (siamois)	2
**Ostéochondrodysplasie**	Achondroplasie	2
**Total**		**37**

La répartition des patients par tranche d'âge et par catégorie de maladies rares: retrouvait une fréquence par grand groupe de maladie rare qui diminuait avec l'âge. Il y avait 20 patients de moins de 5 ans; et 28 de moins de 10 ans. Un seul patient constituait la tranche d'âge la plus élevée, comprise entre 20 et 25 ans (Tableau 3). L'examen clinique retrouvaient des manifestions faciales dans toutes les catégories de maladies rares de notre série. Les lésions faciales prédominantes étaient les hamartomes faciaux (11 cas sur 14 soit 78,5%) dans les hamartoses. Cependant, un nez court, des lèvres épaisses ou des lèvres fines étaient présents diversement dans les autres groupes de maladies rares.

**Tableau 3:** Répartition des patients par tranche d’âge et par groupe de maladies rares

Groupes de maladies	Tranches d’âge					Totaux
	[1-5]	[5-10]	[10-15]	[15-20]	[20-25]	
Hamartoses	5	4	3	1	1	14
Maladies neuromusculaires	2	2	1	2	-	7
Anomalies du développement du cerveau	6	-	-	1	-	7
Retards psychomoteurs syndromiques	3	2	-	-	-	5
Syndromes polymalformatifs	2	-	-	-	-	2
Ostéochondrodysplasies	2	-	-	-		2
**Totaux**	**20**	**8**	**4**	**4**	**1**	**37**

**Figure 2:**
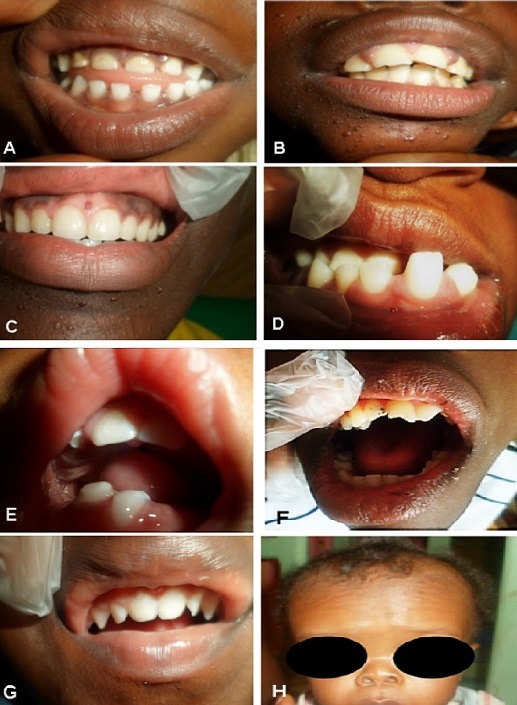
A) hypoplasie de l'émail, dysharmonie dento-maxillaire par excès de place et microdontie sur sclérose Tubéreuse de Bourneville; B) hamartomes faciaux, hypoplasie de l'émail et hyperplasie gingivale sur sclérose Tubéreuse de Bourneville; C) hamartomes facial et gingival sur sclérose tubéreuse de Bourneville; D) macrodontie des blocs deux et trois, inversion de l'articulé dentaire et disto-version de la 32 chez un patient avec syndrome de Protée; E) cas d'holoprosencéphalie avec hypodontie et palais ogival; F) déviation du chemin d'ouverture buccal et usure dentaire sur dystonie deformans; G) cas du syndrome de Wolf Hirchhorn avec hypoplasie de l'émail et incisives latérales et canines conoïdes; H) cas d'achondroplasie avec nez court

Les manifestations bucco-dentaires observées: elles comportaient des atteintes des tissus mous et bases osseuses, des atteintes dentaires en denture temporaire ou en denture permanente, des manifestations fonctionnelles. Concernant les bases osseuses et tissus mous, les anomalies les plus rencontrées variaient d'une maladie rare à l'autre. Dans les hamartoses, il s'agissait d'hamartomes de la muqueuse gingivale (36%); dans les maladies neuromusculaires, c'était l'hypertrophie de la langue, des fasciculations de la langue et l'hypersialie avec trois cas chacun (43%); un palais ogival était plus fréquent avec trois cas chacun dans les retards psychomoteurs syndromiques et anomalies de développement du cerveau. Une langue basse et un palais ogival avec deux cas chacun étaient retrouvés dans les ostéochondrodysplasies. Les lésions les moins rencontrées étaient majoritairement de l'ordre d'un cas dans chaque groupe de maladies rares. Les atteintes dentaires retrouvées étaient diverses. Elles allaient des pathologies carieuses aux anomalies dentaires de nombre, position, forme, structure et taille. Les manifestations dentaires majeures étaient les suivantes: anodontie, hypodontie, microdontie, dentition conique, dentition espacée, diastème antérieur, encombrement dentaire antérieur, usure de l'émail dentaire (Tableau 4). Une association significative a été retrouvée, entre le type de denture et certaines lésions dentaires. Il s'agissait des anomalies de structure (p = 0,001) et de nombre (p = 0,018). En denture temporaire, l'hypodontie était l'anomalie de nombre la plus récurrente, avec les fréquences d'apparition variables: hamartoses (14,2%), maladies neuromusculaires (50%), anomalies de développement du cerveau (40%), syndromes polymalformatifs (50%), ostéochondro-dysplasie (100%). En denture permanente, la carie dentaire était retrouvée dans tous les groupes de maladies rares. Les détails des anomalies dentaires retrouvées sont rapportés dans le Tableau 4. Quelques images des anomalies rencontrées sont illustrées à la Figure 2.

**Tableau 4:** Répartition des anomalies dentaires et atteintes infectieuses en denture temporaire

Groupes de maladies	Signes cliniques	Effectif	Pourcentage (%)
**Hamartoses**	Dentition conoïde	2	28.5
	Macrodontie	3	42.8
	Rotation	1	14.2
	Hypodontie	1	14.2
	Caries	1	14.2
	Encombrement antérieur	2	18.5
	DDM* par excès de place	4	57.1
**Maladies neuromusculaires**	Hypodontie	1	50
	Dentition conoïde	1	50
	DDM par excès de place	2	100
**Anomalies de développement du cerveau**	Hypodontie	2	40
	Microdontie	2	40
	DDM par excès de place	2	40
	Dentition conoïde	2	40
	Béance antérieure	2	40
	Amélogenèse imparfaite	1	20
**Retards psychomoteurs Syndromiques**	Dentition conoïde	2	50
	Encombrement antérieur	2	50
	Amélogenèse imparfaite	2	50
	Usure de l’émail dentaire	2	50
**Syndromes polymalformatifs**	Hypodontie	1	50
**Ostéochondrodysplasie**	Anodontie	2	100

* DDM : Dysharmonie Dento-Maxillaire

**Manifestations fonctionnelles observées**: nous avons noté une association significative entre la présence d'anomalies oro faciales fonctionnelles et les groupes de maladies. Cette association concernait la mastication difficile (p = 0,023), succion linguale/digitale (p = 0,033). Aussi, trois patients avaient une hyper-salivation, il s'agissait des patients atteints du syndrome de Rett et de syndrome de l'X fragile. Par ailleurs, des cas de troubles phonatoires ont été observés dans les pathologies suivantes: sclérose tubéreuse de Bourneville (neuf cas sur neuf), syndrome de Protée (un cas sur trois), et tous les patients du groupe comportant des anomalies neuromusculaires (dystrophie musculaire de Duchenne, amyotrophie spinale infantile, dystonie deformans) (Tableau 5).

**Tableau 5:** Répartition des anomalies orales fonctionnelles selon le groupe de maladie

Anomalies fonctionnelles	Groupes de maladies*						Valeur p
	Groupe 1 N (%)	Groupe 2 N (%)	Groupe 3 N (%)	Groupe 4 N (%)	Groupe 5 N (%)	Groupe 6 N (%)	
Déglutition atypique	3 (21)	3 (43)	4 (57,1)	2 (40)	- (-)	- (-)	0,371
Ventilation mixte	2 (14,2)	2 (28,5)	4 (57,1)	- (-)	- (-)	- (-)	0,35
Mastication difficile	2 (14,2)	4 (57,1)	5 (71,4)	3 (60)	- (-)	2 (100)	0,023
Phonation difficile	10 (71,4)	7 (100)	5 (71,4)	5 (100)	- (-)	2 (100)	0,16
Anomalies de succion	3 (21,4)	1 (14,2)	3 (43)	3 (60)	2 (100)	1(50)	**0,033**
Parafonctions	2 (14,2)	3 (43)	2 (28,5)	2 (40)	- (-)	- (-)	0,87

* **Groupe 1:** hamartoses ; **Groupe 2:** maladies neuromusculaires; **Groupe 3:** anomalies du développement du cerveau; **Groupe 4:** retards psychomoteurs syndromiques; **Groupe 5:** syndromes polymalformatifs ; **Groupe 6:**ostéochondrodysplasies

## Discussion

Cette étude est la première du genre, portant sur les manifestations buccodentaires chez les sujets porteurs de maladies rares au Cameroun. Il en ressort que celles-ci touchent pareillement les deux sexes. Toutefois, certaines maladies spécifiques prédominent chez les sujets d'un sexe ou de l'autre. Concernant l'accès à l'éducation, 62,2% des participants n'étaient pas scolarisés. En effet dans notre contexte, des barrières existent à la scolarisation des enfants handicapés ou à besoins éducatifs particuliers. Celles-ci sont diverses et incluent l'insuffisance d'écoles et éducateurs spécialisés, leur coût prohibitif, l'absence de subventions aux familles affectées, la lenteur à l'implémentation de l'éducation inclusive pourtant décidée par les pouvoirs publics, et qui prévoit l'intégration de cette catégorie d'enfants dans des écoles ordinaires. Ces résultats sont corroborés par ceux de Lamonica *et al*. en 2011, et Hall en 2006, qui rapportaient naturellement dans leurs séries une déficience intellectuelle, un retard mental et des difficultés d'apprentissage dans différentes maladies rares [7,8]. La provenance des patients de 8 des 10 régions de notre pays, rappelle le rôle d'hôpitaux de référence joués par les deux sites de notre étude. Quoique disposant de plusieurs compétences médicales et paramédicales, ce ne sont pas toutefois des centres labellisés maladies rares. Certes, la moitié des patients (51,4%) étaient des ressortissants de la région de l'Ouest Cameroun. Ceci suggère l'existence d'une différence ethnique en termes de prévalence, comme rapporté dans d'autres pays, Romitti *et al*. [9] aux USA… Toutefois dans notre cas, en l'absence d'études socio-anthropologiques approfondies et génétiques avec recherche de consanguinité, on ne peut pas tirer de conclusions hâtives.

### Accès à la génétique et aux soins bucco dentaires

La consultation de génétique est cruciale dans ces pathologies à grande majorité génétiques [10]. Cependant, seuls 21,7% de nos sujets avaient bénéficié de l'expertise d'un généticien. Cette situation est identique aux trouvailles de Rakotoarison à Madagascar [11]. Cependant, Boy-Lefèvre en France [12] dans son étude, incluait l'examen génétique à la première étape de la prise en charge des maladies rares. Cette différence serait liée à la rareté des généticiens et de laboratoires de génétiques dans notre contexte, et dans tous les pays en voie de développement. L'accessibilité aux tests génétiques est variable dans la littérature [13-17]. Avec des taux moins bons au sein de groupes vulnérables. La plupart des tests génétiques dans notre série avaient été réalisés grâce à la coopération avec ALMOHA appuyée par une équipe de généticiens français. La consultation d'un spécialiste bucco-dentaire avait été faite par seulement 13,6% des patients de notre série. Ce qui s'expliquerait par un manque de sensibilisation ou d'intérêt des parents à amener leurs enfants au contrôle annuel de routine chez le médecin bucco-dentaire [18]. Cette situation est également déplorée dans diverses études au sein de population vulnérables ou à besoins spécifiques; souvent en rapport avec une insuffisance de ressources financières ou une marginalisation [18-21]. Hasnaa en 2009 [22] montrait dans son étude que le traitement des affections buccodentaires chez des sujets atteints de déficience physique, alourdissaient le coût de leur prise en charge, alors qu'ils ne bénéficiaient d'aucune aide socio-économique.

### Trouvailles cliniques riches et diverses

Cliniquement, l'hamartome facial, représentait 78,5% des lésions de la peau dans le groupe des hamartoses. De nombreux travaux aux Etats Unis d'Amérique, au Japon, en France, et au Maroc rapportaient une manifestation identique au sein de leurs populations d'étude [22-27]. Le nez court était présent dans les anomalies de développement du cerveau, les syndromes polymalformatifs et les ostéochondrodysplasies, avec des pourcentages respectifs de 71,1% et 100%. Berry *et al*. aux Etats Unis d'Amérique [28], trouvaient le même phénotype, chez 50% des membres d'une famille, souffrant d'holoprosencéphalie. Stephen en 2005 [29], ainsi que Al-Saleem *et al* en 2010 [30], au sujet de patients achondroplasiques décrivaient un pont nasal déprimé, avec diminution du calibre de leurs voies respiratoires et un nez plat. Les hamartomes des muqueuses gingivales étaient présents à 36% dans le groupe des hamartoses à l'examen endobuccal. Plusieurs résultats corroborent les nôtres [26,27], chez des patients atteints de sclérose tubéreuse de Bourneville et de neurofibromatose. En effet, la dysplasie des tissus endo-buccaux, est associée à un trouble de production de certaines protéines régulant la croissance tissulaire. Nous avons également retrouvé un angiome palatin, chez un patient de notre échantillon, atteint du syndrome de Sturge Weber. Un cas identique avait été rencontré dans l'étude de Gill *et al*en 2010 [31]. Ils trouvaient un angiome hyperplasique au niveau de la gencive. Dans les maladies neuromusculaires, 43% des patients atteints de dystrophie musculaire de Duchenne avaient une hypertrophie de la langue. Wonkam au Cameroun [5] et Renard *et al*. en France [32] retrouvaient respectivement 2 et 3 cas d'hypertrophie linguale chez des patients atteints de cette même pathologie. Deux cas d'amyotrophie spinale infantile, présentaient des fasciculations linguales; ces manifestations font partie du tableau clinique habituel de cette affection. Iannaccone aux Etats Unis d'Amérique [33], les avait retrouvés chez 56% des patients de sa série. Les anomalies liées aux malformations des bases osseuses, étaient présentes dans le groupe de patients avec retards psychomoteurs syndromiques. En effet, 60% avaient un palais ogival dans les syndromes de X fragile et de Wolf Hirchhorn. Lamonica et Nieminen avaient retrouvé des résultats similaires [8,34]. Nos 2 cas d'achondroplasie avaient une langue basse. Pourtant, Stephen en Afrique du Sud [29] et Al-Saleem en Arabie Saoudite [30], avaient rapporté une macroglossie chez la totalité des cas similaires. Cette différence, serait due à l'écart d'âge des patients. Mais d'autres facteurs sont à considérer.

### Anomalies dentaires en denture temporaire

Concernant les anomalies dentaires, en denture temporaire, 42,8% des patients avec hamartose étaient en macrodontie. Turner en 2004 [25] retrouvait cette anomalie dentaire de forme chez 18 patients avec le syndrome de Protée, et l'avait qualifiée de dysplasie dentaire. Tous nos patients achondroplasiques présentaient une anodontie à plus d'un an. Pourtant, certains auteurs rapportaient une denture complète chez des adolescents achondroplasiques [29,30]. Une probable carence en micronutriments dans notre série, ou l'âge différent des sujets pourraient expliquer ces disparités. Un des deux cas d'holoprosencéphalie avait une incisive centrale unique mandibulaire, l'autre présentait une dysharmonie dento-maxillaire par excès de place, sauf au niveau des incisives centrales mandibulaires, qui étaient bien centrées et isolées des autres dents. Au contraire, en Australie et aux Etats Unis d'Amérique, des études retrouvaient plutôt une association entre l'holoprosencéphalie et la présence d'une incisive centrale maxillaire unique [7,28]. L'hétérogénéité raciale des populations d'étude, pourrait expliquer la différence entre les sites de défaut de la ligne médiane. Les deux cas d'amyotrophie spinale infantile présentaient une dysharmonie dento-maxillaire par excès de place. Messina *et al.* en 2008 [35] retrouvaient une anomalie similaire auprès de 34 patients. La faiblesse musculaire faciale, serait à l'origine de l'élargissement de l'arcade dentaire, et de ces malpositions dentaires. Concernant les quatre cas de retards psychomoteurs syndromiques, 2 présentaient une amélogenèse imparfaite. L'un était atteint du syndrome de Wolf Hirchhorn, et l'autre souffrait d'un syndrome génétique et cardiaque dont le nom n'était pas encore précisé. Ce résultat est en accord avec celui de Nieminen *et al*. [34] en Finlande à propos du syndrome de Wolf Hirchhorn. Cependant, les patients de son étude étaient en denture permanente.

### Anomalies dentaires en denture permanente

Au sujet de la denture permanente, l'amélogenèse imparfaite était aussi observée chez 40% des patients souffrant d'hamartose. Ce résultat est similaire à celui de Salhi *et al*. Au Maroc à propos de 12 cas de sclérose tubéreuse de Bourneville [36]. Ce défaut de formation de l'émail serait lié à un trouble de fonctionnement des facteurs de croissance et de maturation des tissus minéralisés de la dent. Les patients atteints de maladies neuromusculaires avec une denture permanente, souffraient à 75% de carie dentaire. Engvall en Suède [37] constatait dans ses travaux, que les patients atteints de maladies neuromusculaires avaient plus de caries que ceux du groupe témoin. Il justifiait ce résultat par la diminution de la capacité à maintenir une bonne hygiène bucco-dentaire chez ces patients, liée à leur déficit moteur. La supplémentation systématique en fluor est souvent absente dans cette catégorie sociale.

### Des difficultés fonctionnelles

Un lien significatif existait entre les difficultés à la mastication et tous les groupes de maladies rares. En 2015, Sjogreen et Messina en 2008 [24,35] rapportaient les mêmes difficultés pour s'alimenter chez 20% de ses patients. Enfin, la succion de la langue ou des doigts, de même que des stéréotypies manuelles et comportements autistiques étaient aussi significativement liée aux retards psychomoteurs syndromiques. Ce lien est similaire à celui de plusieurs auteurs, dont les patients étaient atteints des syndromes de Rett et de Wolf Hirchhorn. Nous n'avons pas observé de dents de Hutchinson. Des dyspraxies bucco-linguo-faciales sont assez courantes au cours des affections neurodégénératives et autres retards syndromiques ou non. Elles comportent l'incontinence salivaire, des difficultés de succion déglutition ou manipulation du bol alimentaire par la langue, un retard de passage à une alimentation solide, des fausses routes fréquentes… Par ailleurs, la limitation de l'ouverture de la bouche, la fatigue musculaire accrue des muscles masticatoires et la diminution du tonus musculaire, une inefficacité des lèvres et de la langue, peuvent être plus marqués dans certaines maladies rares… Cette situation peut contribuer à la dénutrition souvent observée chez certains de ces patients. Sjögreen *et al* retrouvaient dans leurs travaux [24], des troubles de la salivation au sein d'une population atteinte du syndrome de Rett (80%) et du syndrome de l'X fragile (40%), résultats corroborant les nôtres. Nos résultats relèvent des difficultés phonatoires chez les patients des groupes de maladies liées aux retards psychomoteurs syndromiques, anomalies neuromusculaires et anomalies de développement du cerveau, similitudes observées par Sjögreen *et al* au sujet des pathologies suivantes: syndrome de Rett, amyotrophie spinale infantile de type I. Plus de 70% des patients atteints de sclérose tubéreuse dans, une méta-analyse en Suède, souffraient aussi de dysfonctionnement phonatoire, comme ceux de notre population d'étude souffrant de la même pathologie. La détérioration de la continence salivaire et de la phonation est probablement liée au déficit de coordination des muscles et nerfs oro-faciaux et au retard psychomoteur généré par les troubles neuromusculaires des patients atteints de maladies rares [32,38-40].

**Limitations**: absence de registre national de maladies rares dans notre pays, et de centres labellisés maladies rares pouvant permettre un recrutement plus important, et participer à la surveillance épidémiologique de ces affections.

## Conclusion

Au terme de cette étude, nous affirmons que les maladies rares sont présentes dans notre contexte. Diverses manifestations orales sont souvent associées; anomalies dentaires de forme, taille, nombre, structure, cavité buccale, avec des répercussions fonctionnelles, nutritionnelles, respiratoires… Celles-ci s'ajoutent aux problèmes buccodentaires classiques retrouvés même chez leurs pairs normotypiques: caries, parodontopathies… Nos trouvailles confirment le besoin en soins buccodentaires préventifs et curatifs dans cette catégorie de patients. Une sensibilisation des familles et des professionnels de santé est à faire de même qu'une augmentation de l'offre des soins bucco-dentaires. Ces soins devraient être intégrés dans le paquet minimum des activités des formations sanitaires et rendus gratuits ou financièrement accessibles à ces patients en situation de handicap ou présentant des besoins spécifiques.

### État des connaissances actuelles sur le sujet

- Des manifestations bucco-dentaires peuvent exister au cours de certaines maladies rares, et impacter négativement sur la santé bucco dentaires et au niveau fonctionnel;
- Des soins spécifiques sont souvent nécessaires chez ces parents;
- La question est insuffisamment étudiée en Afrique.

### Contribution de notre étude à la connaissance

- Évaluation pionnière du problème dans notre contexte;
- Cette étude nous apporte des éléments de plaidoyer pour l'amélioration objective de l'offre de soins à cette catégorie de patients souvent marginalisés.

## Conflits d’intérêts

Les auteurs ne déclarent aucun conflit d’intérêts.

## Remerciements

À l'Association pour la lutte contre les maladies rares, les maladies orphelines et le handicap en Afrique (ALMOHA), pour l'appui technique et l'expertise génétique ayant permis de préciser bien avant cette étude, le diagnostic de plusieurs de nos patients. Au Pr Agnès Bloch-Zupan, de la Faculté de chirurgie dentaire de l'Université de Strasbourg, pour sa contribution dans la conception et l'ébauche de l'étude, l'analyse et l'interprétation des données, la lecture et la correction du travail.
